# The importance of ganglion cell complex investigation in myopic patients

**Published:** 2017

**Authors:** Maria Veronica Strehaianu, Dana Dascalescu, Catalina Ionescu, Miruna Burcel, Vasile Potop, Catalina Corbu

**Affiliations:** * Clinical Ophthalmology Emergency Hospital Bucharest, Romania; ** Oftaclinic Clinic Bucharest, Romania

**Keywords:** ganglion cell complex, glaucoma, myopia

## Abstract

Glaucoma is an optic neuropathy that affects the ganglion cell complex in all its components: cell bodies, dendrites, and axons, the dendritic arbor being the first one damaged. This is the reason why the thickness of the ganglion cell and internal plexiform layers can be taken into account as an early predictor of the glaucomatous changes, along with the retinal nerve fiber layer (RNFL) thickness. However, due to disc tilting and peripapillary atrophy, the RNFL evaluation may be prone to errors in myopic patients.

We presented the cases of two myopic patients, who, after a routine examination, were identified as glaucoma suspects. The Optical Coherence Tomography (OCT) scan revealed a nerve fiber loss which was not confirmed by the ganglion cell complex scan. Thereafter we manually adjusted the optic disc margins according to the patients’ myopic changes and this time the retinal nerve fiber layer was also normal. We observed that the ganglion cell complex evaluation led to fewer errors than the retinal nerve fiber layer evaluation, particularly in front of a myopic patient. Nevertheless, various investigations should be considered in the attempt to issue a diagnosis of glaucoma.

## Introduction

Glaucoma is considered the second cause of blindness globally, after cataracts, but unlike cataracts, glaucoma causes irreversible damage [**1**]. 

Glaucoma induces permanent structural changes in the optic nerve, which translates into permanent functional changes, decreased visual acuity, and specific losses in the visual field [**2**]. 

Glaucoma affects the ganglion cell complex in all its components: cell bodies, dendrites, and axons. The ganglion cell is a large complex structure that extends from the retina to the midbrain [**3**]. In glaucoma, the dendritic arbor is the first one affected due to mitochondrial changes, followed by the cell body and lastly the axon [**4**]. The ganglion cell layer thickness is measured over the macular region, taking into consideration the fact that over 50% of the ganglion cells are localized [**5**]. As a consequence, the Optical Coherence Tomography (OCT) scan for the ganglion cell complex includes ganglion cell layer (GCL), internal plexiform layer (IPL) and nerve fiber layer (NFL) [**6**]. Given its sensitivity, specificity and reproducibility when investigating the structure of the retina, providing the possibility of observing the progression of the structural changes, the OCT scan could be relied on in our attempt to issue an early diagnosis of glaucoma (before visual field loss) [**7**,**8**]. Understanding the cell death mechanism, we know that the internal plexiform layer is the first one affected, followed by the ganglion cell layer and ultimately the nerve fiber layer. This is the reason why we can take the thickness of the ganglion cell and internal plexiform layers into account as an early predictor of the glaucomatous changes, rather than the retinal nerve fiber layer (RNFL) thickness that only depicts the axonal death [**3**]. 

Given the fact that the main goal in the treatment of a glaucomatous patient is avoiding the visual field loss and that approximately 40% of the axons need to be lost in order to detect an early threshold visual field defect, we should consider ganglion cell complex scan especially in myopic patients [**9**,**10**], knowing the fact that myopia has been reported as a risk factor for glaucoma [**11**]. In these particular situations, the optic nerve head examination may be difficult due to the specific changes: optic nerve tilting and peripapillary atrophy thus the optic disc margins cannot be clearly defined by the OCT and the RNFL evaluation may be prone to errors [**3**]. 

## Case report

We discussed the cases of two myopic patients who presented for a routine examination.

**Case 1**/

Case 1 was a 26-year-old female patient. At presentation, her best-corrected visual acuity was 1 for both eyes. Goldman intraocular pressure was 23 mmHg in the right eye (RE) and 22 mmHg in the left eye (LE). The central corneal thickness was 600 microns in both eyes. Regarding the corneal biomechanical properties, the ocular response analyzer revealed IOPcc 20,6 mmHg, IOPg 22,8 mmHg, CH 10,2, CRF 10,1 in the RE and IOPcc 20,2 mmHg, IOPg 22,1 mmHg, CH 10,5, CRF 10,3 in the LE. Gonioscopy showed an open angle in both eyes. Fundus exam showed a cup disc ratio of 0,4 in both eyes. 

**Fig. 1:**
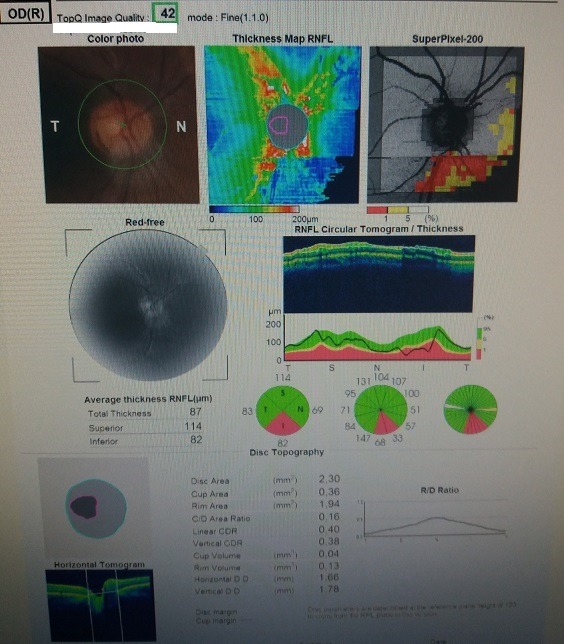
Right eye OCT

**Fig. 2:**
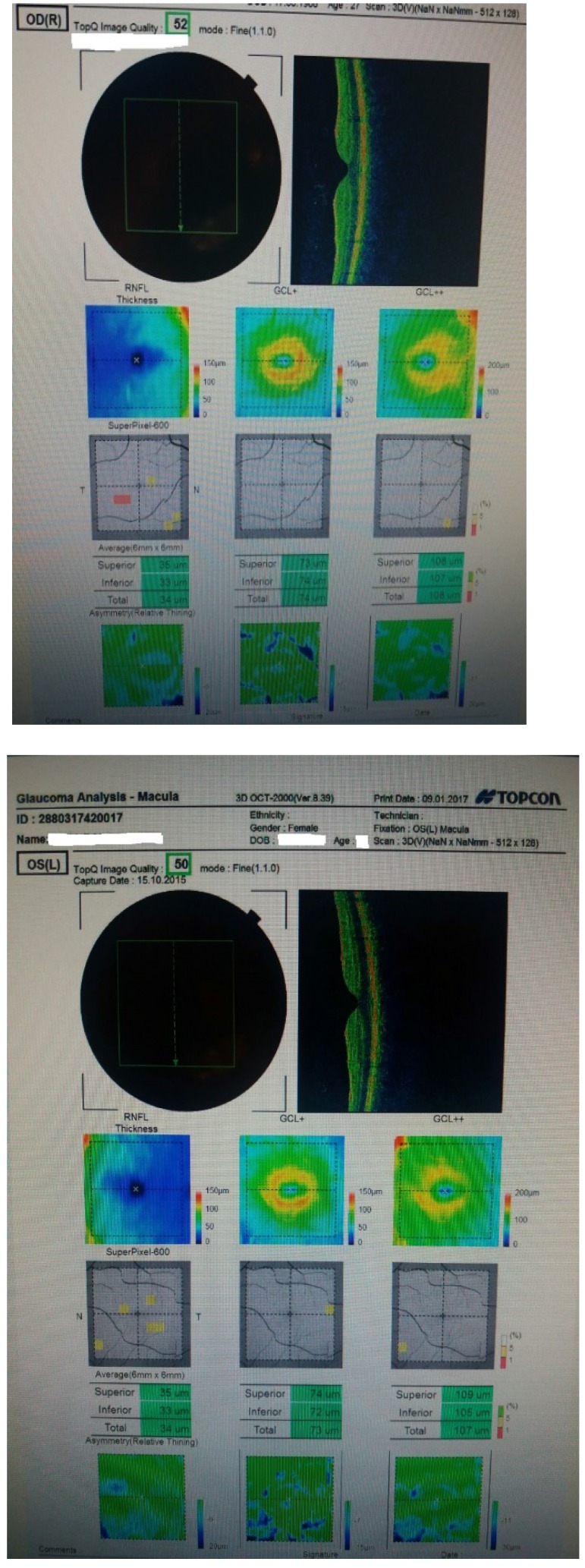
GCC examination; warm colors represent thicker areas, cold colors indicate thinner areas

We performed an optical coherence tomography (**Fig. 1**), that showed a severe loss of nerve fibers in the inferior quadrant in the right eye and no loss in the left eye. We continued by assessing the ganglion cell complex that showed no reduction or thinning (**Fig. 2**). Visual field examination was normal in both eyes.

In front of these two contradictory exams, we started to reevaluate the case and redid all examinations taking into account our patients’ peculiarities. This time, we paid more attention to the disc margins and then we manually adjusted the optic disc margins considering the peripapillary atrophy (**Fig. 5**). After the adjustment, the result was in concordance with the ganglion cell complex measurement and with the other performed exams.

**Case 2**

Case 2 was a 25-year-old male patient. At presentation, his best-corrected visual acuity was 1 in both eyes. With the Goldman applanation tonometer, the intraocular pressure was measured as 20 mmHg in the right eye and respectively 21 mmHg in the left eye. The central corneal thickness was 575 microns in the right eye and 579 microns in the left eye. Ocular response analyzer revealed IOPcc 20,1 mmHg, IOPg 20,4 mmHg, CH 10,8, CRF 10,3 in the RE and IOPcc 21,4 mmHg, IOPg 21,7 mmHg, CH 10,6, CRF 10,2 in LE. Gonioscopy showed an open angle in both eyes. As for the fundus exam, the cup disc ratio was symmetrical and was appreciated as 0,6-0,7.

**Fig. 3:**
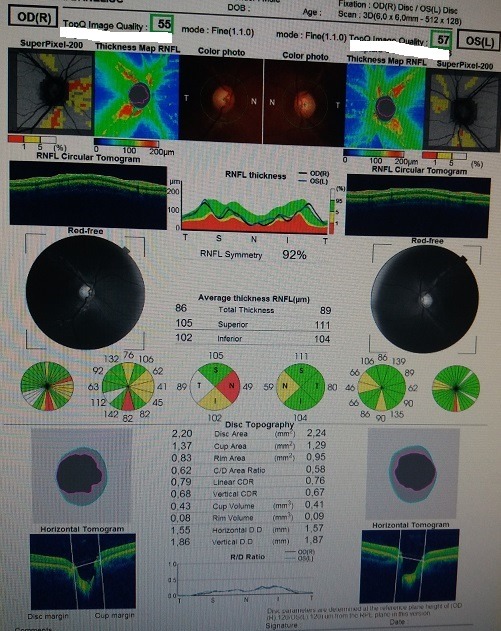
Right and left eye OCT

**Fig. 4:**
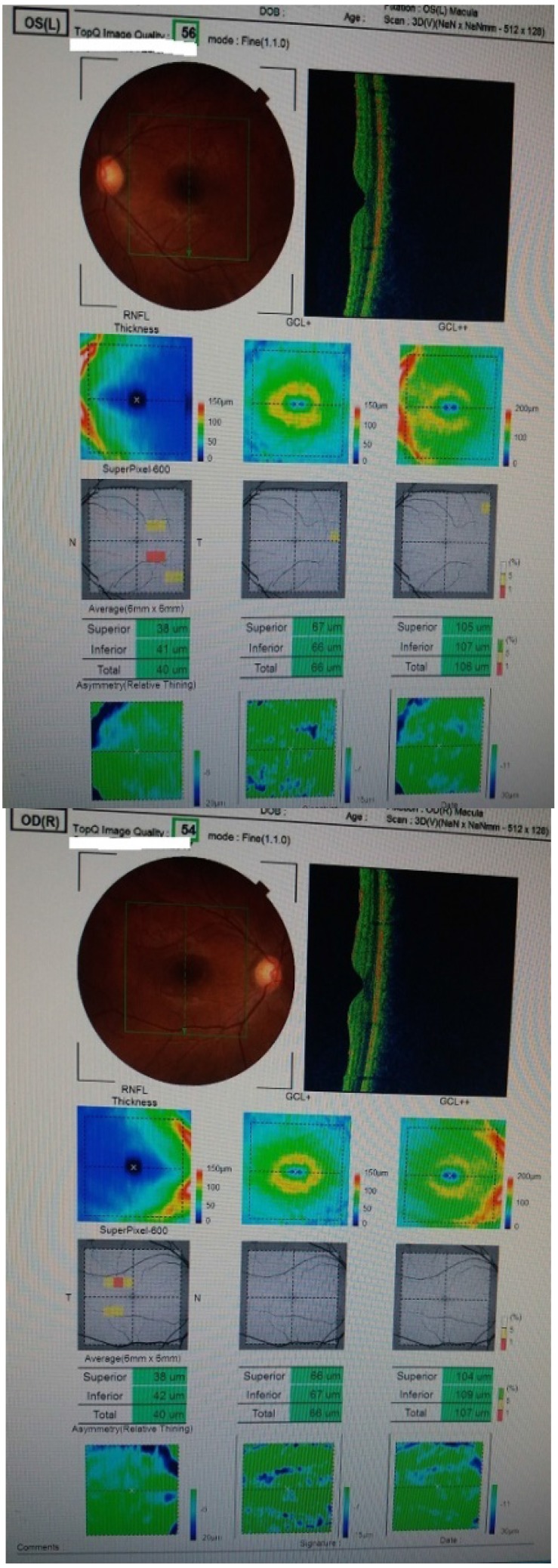
GCC examination; warm colors represent thicker areas, cold colors indicate thinner areas

We performed an optical coherence tomography (**Fig. 3**) that revealed a severe loss of nerve fibers in the nasal quadrant and a moderate nerve fiber loss in the inferior quadrant for the right eye and a moderate nerve fiber loss in the nasal quadrant in the left eye. We continued by assessing the ganglion cell complex, which showed no reduction or thinning (**Fig. 4**).

**Fig. 5:**
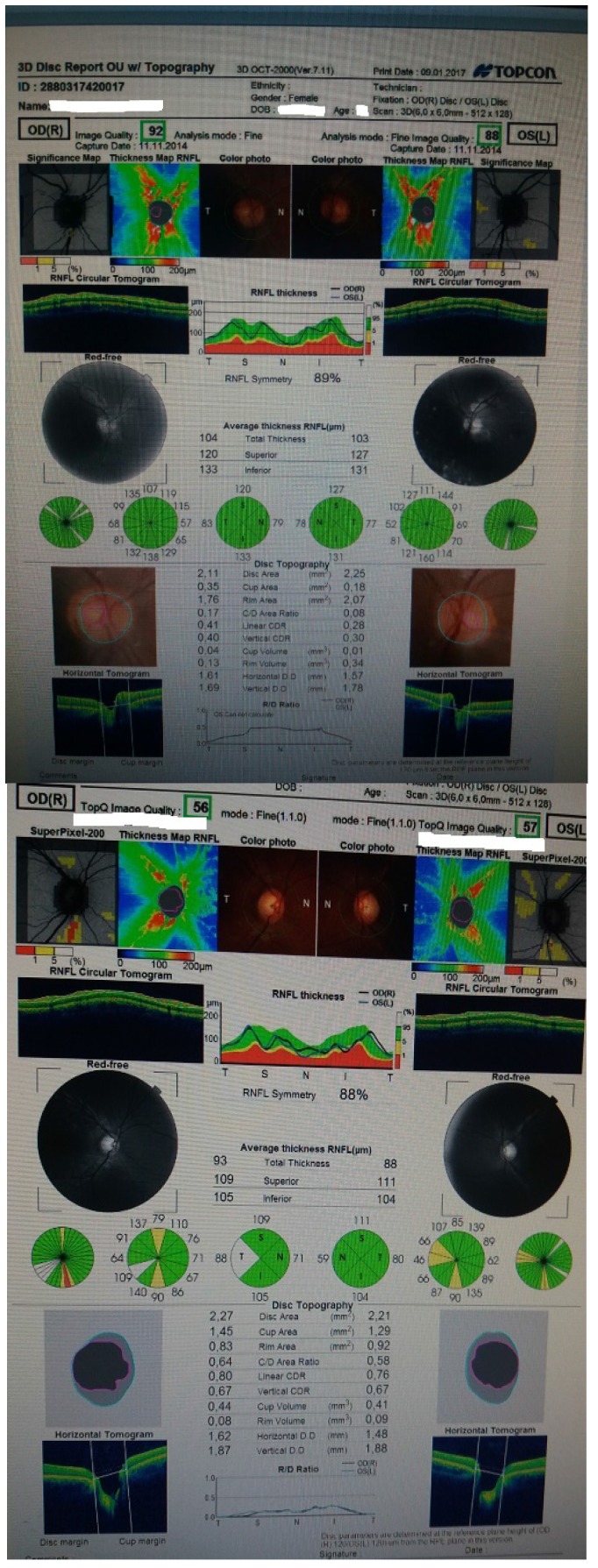
Case 1 and case 2 OCT after adjusting the optic disc margins

This time we also noticed the fact that the two investigations were out of line with each other, so we carefully adjusted the margins of the optic disc according to the patients’ myopic changes and performed another OCT that did not show any loss of nerve fibers (**Fig. 5**).

## Discussions

We observed that myopia could influence the results of the OCT scan, revealing a false thinner nerve fiber layer. As known, the OCT database does not include myopic individuals, so we have to rely on other investigations in order to confirm the glaucoma diagnosis [**11**]. 

After assessing the ganglion cell layer, we discovered that there were no changes suggesting a nerve fiber loss in some of these patients. This is the reason why we manually adjusted the contour of the optic disc and after doing so, the retinal nerve fiber layer showed no pathological findings. These observations are supported by some data in literature that showed some significant associations between RNFL thickness and myopia [**11**]. Also, Budenz et al. described a significant thinning of the RNFL with the increase of the axial length [**12**]. 

Furthermore, we should take into consideration the careful evaluation of the ganglion cell layer thickness, given the fact that this particular investigation provides information mostly about the macular region and is prone to less false positive results than the RNFL, especially in front of a myopic patient [**5**]. Besides, in their study, Sezgin et al. noted that there was no significant correlation between the ganglion cell complex layer and the axial length in moderate and high myopia groups [**13**]. 

Kim’s study showed that there was a significant difference between the values of the ganglion cell complex in normal patients and those with preperimetric glaucoma and that scanning the ganglion cell complex offered similar results with the RNFL scan in preperimetric glaucoma patients [**15**].

As various studies reported, we should not rely only on one investigation and only one examination in front of a glaucoma suspect [**11**,**12**,**14**]. Therefore, the patients should be evaluated, using not only the OCT and the ganglion cell complex, but also the 24-2 Humphrey visual field and intraocular pressure, in order to detect any minor change that could lead to an early glaucoma diagnosis.

## Conclusions

The ganglion cell complex can be used for the evaluation of the nerve fiber layer in front of a myopic, glaucoma suspect patient. 

However, in our pursuit of certifying a glaucoma diagnosis, we should not take into consideration only one investigation. 

Additional, randomized studies are necessary in order to confirm the obtained data and for the ganglion cell complex scan to become a universally accepted investigation for the early diagnosis of glaucoma.

**Acknowledgements**

All authors have an equal contribution to and an equal participation in the paper.
